# Supplementary figures and images for: Allelic variation of Escherichia coli outer membrane protein A: Impact on cell surface properties, stress tolerance and allele distribution
Source: PLoS One. 2022 Oct 13;17(10):e0276046. doi: 10.1371/journal.pone.0276046 (PMC9560509; doi:10.1371/journal.pone.0276046)

Supplemental Figure 1

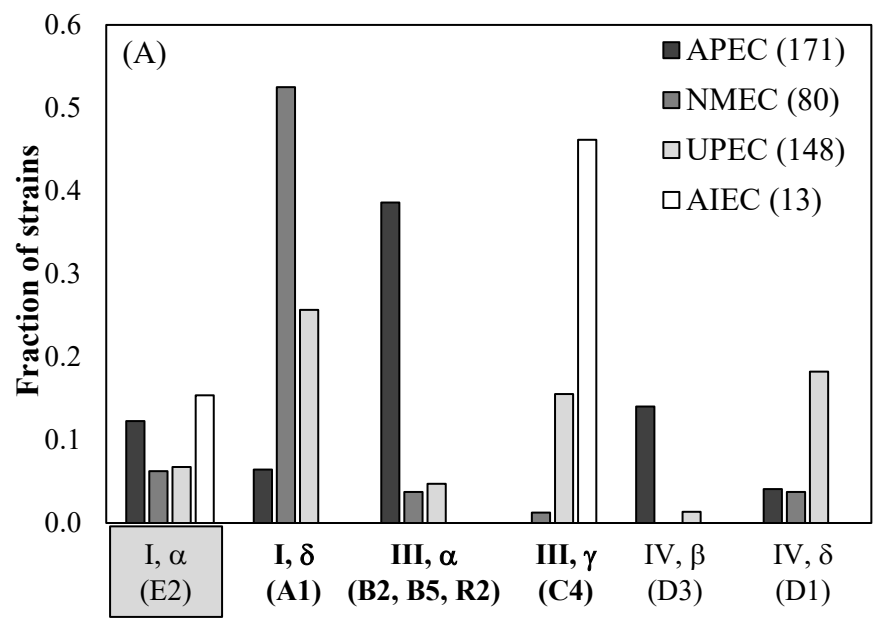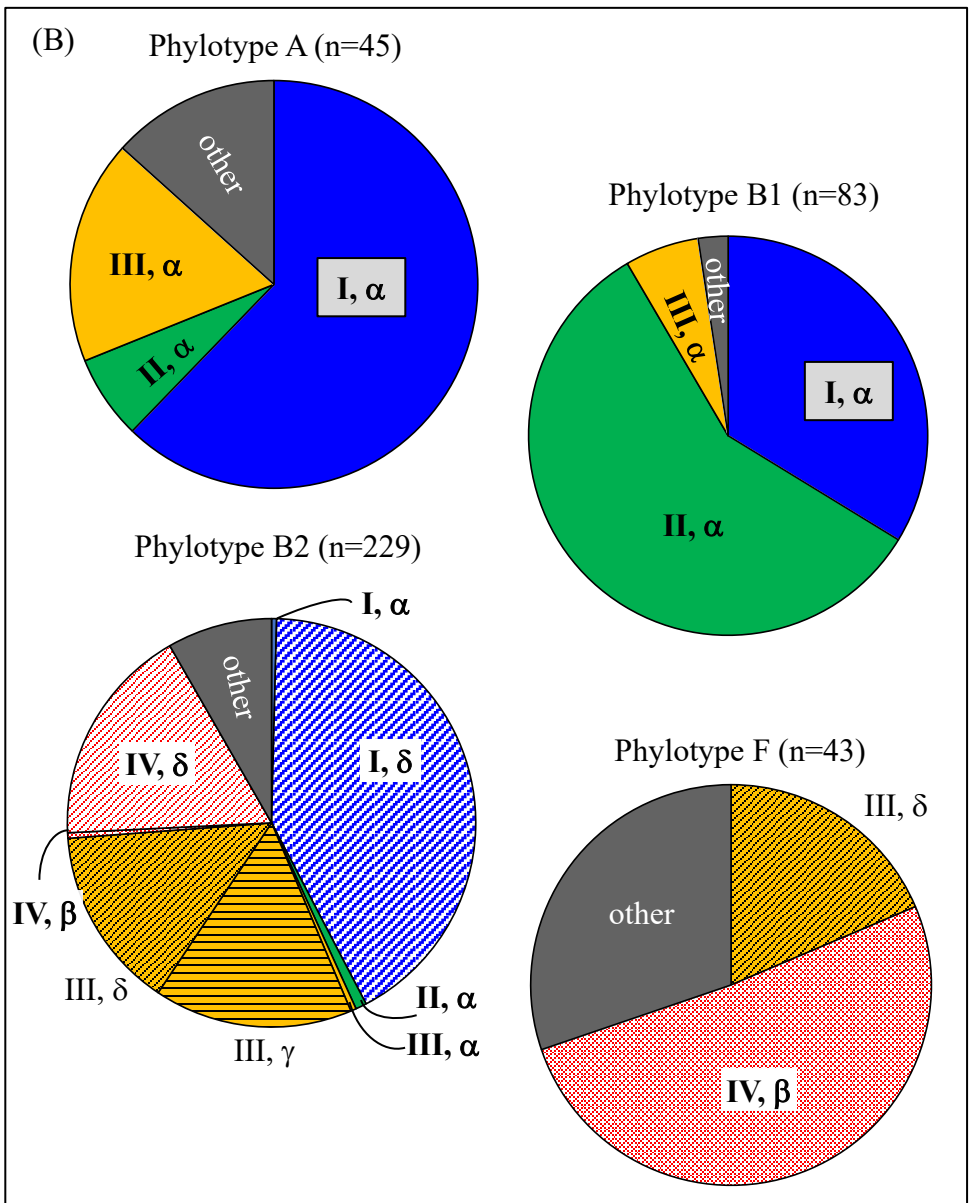

Supplement: S1 Fig — Fisher’s Exact test was used to identify significant differences (two tailed, p<1x10^-5^), as summarized in Table 2 in the main text. Bold font indicates alleles with significant differences, described in Table 2. Terms in parentheses below the x-axis labels are the naming system used by Nielsen et al. (A) Distribution of OmpA alleles among ExPEC isolates. Bold font indicates significant differences. (B) Phylotype distribution among the environmental and ExPEC isolates. Data is only shown for A, B1, B2 and F phylotypes. Color coding indicates outer loop allele (I, blue; II, green; III, gold; IV, red) and fill indicates C-terminal domain (α, solid; β, dotted; γ, horizontal stripes; δ, diagonal stripes). The (I, α) allele is indicated in each figure–this is the allele encoded in K-12 E. coli such as MG1655. (PDF) [file pone.0276046.s001.pdf]
