# Supplementary material for: Allelic variation of Escherichia coli outer membrane protein A: Impact on cell surface properties, stress tolerance and allele distribution
Source: PLoS One. 2022 Oct 13;17(10):e0276046. doi: 10.1371/journal.pone.0276046 (PMC9560509; doi:10.1371/journal.pone.0276046)
Supplement: S1 Table — (PDF) [file pone.0276046.s002.pdf]

Table S1: 100% identity matches to delta allele

Query:
FGQGEAAPVVAPAPAPAPEVQTKHFTLKSDVLFTFNKATLKPEGQAALDQLYSQLSNLDPKDGSVVVLGYTDRIGSDAYNQALSERRAQSVVDYLISKGIPADKISARGMGESNPVTGNTCDNVKQRAALIDCLAPDRRVE
IEVKGIKDVVTQPQA

accession #	note	publication	N-terminal pattern	sequence
ABE06507	UTI89	PUBMED 16585510	I	1 msrspvkdlt vlsrwryswr ilddneaqkm kktaiaiava lagfatvaqa apkdntwytg<br>61 aklgwsqyhd tgfinnngpt henqlgagaf ggyqvnpyvg femgydwlgr mpykgsveng<br>121 aykaqgvqlt aklgypitdd ldvytrlggm vwradtksnv ygknhdtgvs pvfaggveya<br>181 itpeiatrle yqwtnnigda htigtrpdng mlslgvsyrf gqgeaapvva papapapevq<br>241 tkhftlksdv lftfnkatlk pegqaaldql ysqlsnldpk dgsvvvlgyt drigsdaynq<br>301 alserraqsv vdyliskgip adkisargmg esnpvtgntc dnvkqraali dclapdrrve<br>361 ievkgikdvv tqpqa
ABJ00366	APEC O1 O1:K1 lesion of dead turkey with colibacillosis Iowa, USA, 2006	PUBMED 17293413	I	1 mkdltvlsrw ryswrilddn eaqkmkktai aiavalagfa tvaqaapkdn twytgaklgw<br>61 sqyhdtgfin nngpthenql gagafggyqv npyvgfemgy dwlgrmpykg svengaykaq<br>121 gvqltaklgy pitddldvyt rlggmvwrad tksnvygknh dtgvspvfag gveyaitpei<br>181 atrleyqwtn nigdahtigt rpdngmlslg vsyrfgqgea apvvapapap apevqtkhft<br>241 lksdvlftfn katlkpegqa aldqlysqls nldpkdgsvv vlgytdrigs daynqalser<br>301 raqsvvdyli skgipadkis argmgesnpv tgntcdnvkq raalidclap drrveievkg<br>361 ikdvvtqpqa
ADR26297	Escherichia coli O83:H1 str. NRG 857C, associated with Crohn's disease	PUBMED 18312396	IV	1 mlsrwryswr ilddneaqkm kktaiaiava lagfatvaqa apkdntwytg aklgwsqyhd<br>61 tgfinnngpt henqlgagaf ggyqvnpyvg femgydwlgr mpykgsveng aykaqgvqlt<br>121 aklgypitdd ldvytrlggm vwradtksnf dgknhdtgvs pvfaggveya itpeiatrle<br>181 yqwtnnigda htigtrpdng mlslgvsyrf gqgeaapvva papapapevq tkhftlksdv<br>241 lftfnkatlk pegqaaldql ysqlsnldpk dgsvvvlgyt drigsdaynq alserraqsv<br>301 vdyliskgip adkisargmg esnpvtgntc dnvkqraali dclapdrrve ievkgikdvv<br>361 tqpqa
ADN71927	E. coli UM146	PUBMED 21075930	I	1 mlsrwryswr ilddneaqkm kktaiaiava lagfatvaqa apkdntwytg aklgwsqyhd<br>61 tgfinnngpt henqlgagaf ggyqvnpyvg femgydwlgr mpykgsveng aykaqgvqlt<br>121 aklgypitdd ldvytrlggm vwradtksnv ygknhdtgvs pvfaggveya itpeiatrle<br>181 yqwtnnigda htigtrpdng mlslgvsyrf gqgeaapvva papapapevq tkhftlksdv<br>241 lftfnkatlk pegqaaldql ysqlsnldpk dgsvvvlgyt drigsdaynq alserraqsv<br>301 vdyliskgip adkisargmg esnpvtgntc dnvkqraali dclapdrrve ievkgikdvv<br>361 tqpqa
HAN9326727	E. coli M658539, human feces, Australia, 2015	PUBMED 30286803	I	1 mkktaiaiav alagfatvaq aapkdntwyt gaklgwsqyh dtdfinnngp thenqlgaga<br>61 fggyqvnpyv gfemgydwlg rmpykgsven gaykaqgvql taklgypitd dldvytrlgg<br>121 mvwradtksn vygknhdtgv spvfaggvey aitpeiatrl eyqwtnnigd ahtigtrpdn<br>181 gmlslgvsyr fgqgeaapvv apapapapev qtkhftlksd vlftfnkatl kpegqaaldq<br>241 lysqlsnldp kdgsvvvlgy tdrigsdayn qalserraqs vvdyliskgi padkisargm<br>301 gesnpvtgnt cdnvkqraal idclapdrrv eievkgikdv vtqpqa
HAX4482397	E. coli SewageA-083, Japan, 2017	PUBMED 30286803	I	1 mkktaiaiav alagfatvaq aapkdntwyt gaklgwsqyh dtgfinnngp thenqlgaga<br>61 fggyqvnpyv gfemgydwlg rmpykgsven gaykaqgvql taklgypitd dldvytrlgg<br>121 mvwradtksn vygknhdtgv spvfaggvey aitpeistrl eyqwtnnigd ahtigtrpdn<br>181 gmlslgvsyr fgqgeaapvv apapapapev qtkhftlksd vlftfnkatl kpegqaaldq<br>241 lysqlsnldp kdgsvvvlgy tdrigsdayn qalserraqs vvdyliskgi padkisargm<br>301 gesnpvtgnt cdnvkqraal idclapdrrv eievkgikdv vtqpqa
HAX5866669	isolate C92, O25b:H4, human isolate from France	PUBMED 30286803	III	1 mkktaiaiav alagfatvaq aapkdntwyt gaklgwsqyh dtgfipnnsp thenqlgaga<br>61 fggyqvnpyv gfemgydwlg rmpykgdni!n gaykaqgvql taklgypitd dldiytrlgg<br>121 mvwradtkan vpggasfkdh dtgvspvfag gveyaitpei atrleyqwtn nigdahtigt<br>181 rpdngmlslg vsyrfgqgea apvvapapap apevqtkhft lksdvlftfn katlkpegqa<br>241 aldqlysqls nldpkdgsvv vlgytdrigs daynqalser raqsvvdyli skgipadkis<br>301 argmgesnpv tgntcdnvkq raalidclap drrveievkg ikdvvtqpqa
HBB7539254	sewage, Japan (2015)	PUBMED 30286803	III	1 mkktaiaiav alagfatiaq aapkdntwyt gaklgwsqyh dtgfipnngp thenqlgaga<br>61 fggyqvnpyv gfemgydwlg rmpykgdni!n gaykaqgvql taklgypitd dldiytrlgg<br>121 mvwradtkan vpggasfkdh dtgvspvfag gveyaitpei atrleyqwtn nigdahtigt<br>181 rpdngmlslg vsyrfgqgea apvvapapap apevqtkhft lksdvlftfn katlkpegqa<br>241 aldqlysqls nldpkdgsvv vlgytdrigs daynqalser raqsvvdyli skgipadkis<br>301 argmgesnpv tgntcdnvkq raalidclap drrveievkg ikdvvtqpqa
HBH9295071	AUSMDU00034110, human urine, Australia (2019)	PUBMED 30286803	III	1 mkktaiaiav alagfatvaq aapkdntwyt gsklgwsqyh dtgfipnngp thenqlgaga<br>61 fggyqvnpyv gfemgydwlg rmpykgdni!n gaykaqgvql taklgypitd dldiytrlgg<br>121 mvwradtkan vpggasfkdh dtgvspvfag gveyaitpei atrleyqwtn nigdahtigt<br>181 rpdngmlslg vsyrfgqgea apvvapapap apevqtkhft lksdvlftfn katlkpegqa<br>241 aldqlysqls nldpkdgsvv vlgytdrigs daynqalser raqsvvdyli skgipadkis<br>301 argmgesnpv tgntcdnvkq raalidclap drrveievkg ikdvvtqpqa
HAL2078290	E. coli G034, human, Washington state, 2010	PUBMED 30286803	IV	1 mkktaiaiav alagfatvaq aapkdntwyt gaklgwsqyh dtgfinnngp thenqlgaga<br>61 fggyqvnpyv gfemgydwlg rmpykgsven gaykaqgvql taklgypitd dldvytrlgg<br>121 mvwradtksn fdgknhdtgv spvfaggvey aitpeiatrl eyqwtnnigd ahtigtrpdn<br>181 gmlslsvsyr fgqgeaapvv apapapapev qtkhftlksd vlftfnkatl kpegqaaldq<br>241 lysqlsnldp kdgsvvvlgy tdrigsdayn qalserraqs vvdyliskgi padkisargm<br>301 gesnpvtgnt cdnvkqraal idclapdrrv eievkgikdv vtqpqa
HAP3737440	Escherichia coli, AMC_922, human, United Kingdom, 2013	PUBMED 30286803	IV	1 mkktaiaiav alagfatvaq aapkdntwyt gaklgwsqyh dtgfinnngp thenqlgaga<br>61 fggyqvnpyv gfemgydwlg rmpykgsven gaykaqgvql taklgypitd dldvytrlgg<br>121 mvwradtksn fdgknhdtgv spvfaggvey aitpeiatrl eyqwtnnigd ahtigtrpdn<br>181 gllslgvsyr fgqgeaapvv apapapapev qtkhftlksd vlftfnkatl kpegqaaldq<br>241 lysqlsnldp kdgsvvvlgy tdrigsdayn qalserraqs vvdyliskgi padkisargm<br>301 gesnpvtgnt cdnvkqraal idclapdrrv eievkgikdv vtqpqa

Table S1: 100% identity matches to delta allele

HBA5002621	E. coli isol104, human feces, Netherlands, 2013	PUBMED 30286803	IV	1 mkktaiaiav alagfatvaq aapkdntwyt gaklgwsqyh dtgfinnngp thenqlgaga<br>61 fggyqvnpyv gfemgydwlg rmpykgsven gaykaqgvql taklgypitd dldvytrlgg<br>121 mvwradtksn fdgknhdtgv spafaggvey aitpeiatrl eyqwtnnigd ahtigtrpdn<br>181 gmlslgvsyr fgqgeaapvv apapapapev qtkhftlksd vlftfnkatl kpegqaaldq<br>241 lysqlsnldp kdgsvvvlgy tdrigsdayn qalserraqs vvdyliskgi padkisargm<br>301 gesnpvtgnt cdnvkqraal idclapdrrv eievkgikdv vtqpqa
HAX8716775	E. coli ITUmcc122, human urine, France, 2017, subgroup B2	PUBMED 30286803	IV	1 mkktaiaiav alagfatvaq aapkdntwyt gaklgwsqyh dtgfinnngp thenqlgaga<br>61 fggyqvnpyv gfemgydwlg rmpykgsven gaykaqgvql taklgypitd dldvytrlgg<br>121 mvwradtksn fdgknhdtgv spvfaggvey aitpeiatrl eyqwtnnigd artigtrpdn<br>181 gmlslgvsyr fgqgeaapvv apapapapev qtkhftlksd vlftfnkatl kpegqaaldq<br>241 lysqlsnldp kdgsvvvlgy tdrigsdayn qalserraqs vvdyliskgi padkisargm<br>301 gesnpvtgnt cdnvkqraal idclapdrrv eievkgikdv vtqpqa
HBC5707382	E. coli 1D-1122, human isolate, Denmark	PUBMED 30286803	other	1 mkktaiaiav alagfatvaq aapkdntwyt gaklgwsqyh dtgfidnngp thenqlgaga<br>61 fggyqvnpyv gfemgydwlg rmpykgsven gaykaqgvql taklgypitd dldvytrlgg<br>121 mvwradtksn vygknhdtgv spvfaggvey aitpeiatrl eyqwtnnigd ahtidtrpdn<br>181 gmlslgvsyr fgqgeaapvv apapapapev qtkhftlksd vlftfnkatl kpegqaaldq<br>241 lysqlsnldp kdgsvvvlgy tdrigsdayn qalserraqs vvdyliskgi padkisargm<br>301 gesnpvtgnt cdnvkqraal idclapdrrv eievkgikdv vtqpqa
HAJ3443934	Escherichia coli EuSCAPE_FI009	PUBMED 30286803	other	1 mkktaiaiav alagfatvaq aapkdntwyt gaklgwsqyh dtgfinnngp thenqlgaga<br>61 fggyqvnpyv gfemgydwlg rmpykgsvkn gaykaqgvql taklgypitd dldvytrlgg<br>121 mvwradtksn fdgknhdtgv spvfaggvey aitpeiatrl eyqwtnnigd ahtigtrpdn<br>181 gmlslgvsyr fgqgeaapvv apapapapev qtkhftlksd vlftfnkatl kpegqaaldq<br>241 lysqlsnldp kdgsvvvlgy tdrigsdayn qalserraqs vvdyliskgi padkisargm<br>301 gesnpvtgnt cdnvkqraal idclapdrrv eievkgikdv vtqpqa
HAL0437146	E. coli HVH_80_4_2428830_, human urine, Canada, 2013	PUBMED 30286803	other	1 mkktaiaiav alagfatvaq aapkdntwyt gaklgwsqyh dtgfinnngp thenqlgaga<br>61 fggyqvnpyv gfemgydwlg rmpykgsven gaykaqgvql taklgypitd dldvytrlgg<br>121 mvwradtksn iygknhdtgv spvfaggvey aitpeiatrl eyqwtnnigd ahtigtrpdn<br>181 gmlslgvsyr fgqgeaapvv apapapapev qtkhftlksd vlftfnkatl kpegqaaldq<br>241 lysqlsnldp kdgsvvvlgy tdrigsdayn qalserraqs vvdyliskgi padkisargm<br>301 gesnpvtgnt cdnvkqraal idclapdrrv eievkgikdv vtqpqa
HAL6127952	isolate 39, human urine, Minnesota, 1996	PUBMED 30286803	other	1 mkktaiaiav alagfatvaq aapkdntwyt gaklgwsqyh dtgfipnngp thenqlgaga<br>61 fggyqvnpyv gfemgydwlg rmpyngayka qgvqltaklg ypitddldiy trlggmvwra<br>121 dtktnvlgga sfkdhdtgvs pvfaggveya itpeiatrle yqwtnnigda htidtrpdng<br>181 mlslgvsyrf gqgeaapvva papapapevq tkhftlksdv lftfnkatlk pegqaaldql<br>241 ysqlsnldpk dgsvvvlgyt drigsdaynq alserraqsv vdyliskgip adkisargmg<br>301 esnpvtgntc dnvkqraali dclapdrrve ievkgikdvv tqpqa
HAH1656328	eo2582	PUBMED 30286803	other	1 mkktaiaiav alagfatvaq aapkdntwyt gaklgwsqyh dtgfipnngp thenqlgaga<br>61 fggyqvnpyv gfemgydwlg rmpykgdni!n gaykaqgvql taklgypitd dldiytrlgg<br>121 mvwradtkan vpggasfkyh dtgvspvfag gveyaitpei atrleyqwtn nigdahtigt<br>181 rpdngmlslg vsyrfgqgea apvvapapap apevqtkhft lksdvlftfn katlkpegqa<br>241 aldqlysqls nldpkdgsvv vlgytdrigs daynqalser raqsvvdyli skgipadkis<br>301 argmgesnpv tgntcdnvkq raalidclap drrveievkg ikdvvtqpqa
HAH6184449	HICF248, human blood, United Kingdom	PUBMED 30286803	other	1 mkktaiaiav alagfatvaq aapkdntwyt gaklgwsqyh dtgfipnngp thenqlgaga<br>61 fggyqvnpyv gfemgydwlg rmpykgdni!n gaykaqgvql taklgypitd dldiytrlgg<br>121 mvwradtktn vlggasfkdh dtgvspvfag gveyaitpei atrleyqwtn nigdahtigt<br>181 rpdngmlslg vsyrfgqgea apvvapapap apevqtkhft lksdvlftfn katlkpegqa<br>241 aldqlysqls nldpkdgsvv vlgytdrigs daynqalser raqsvvdyli skgipadkis<br>301 argmgesnpv tgntcdnvkq raalidclap drrveievkg ikdvvtqpqa
HAP1838883	Ecoli868, human bile duct fluid, San Diego, CA	PUBMED 30286803	other	1 mkktaiaiav alagfatvaq aapkdntwyt gaklgwsqyh dtgfipnngp thenqlgaga<br>61 fggyqvnpyv gfemgydwlg rmpykgnnin gaykaqgvql taklgypitd dldiytrlgg<br>121 mvwradtkan vpggasfkdh dtgvspvfag gveyaitpei atrleyqwtn nigdahtigt<br>181 rpdngmlslg vsyrfgqgea apvvapapap apevqtkhft lksdvlftfn katlkpegqa<br>241 aldqlysqls nldpkdgsvv vlgytdrigs daynqalser raqsvvdyli skgipadkis<br>301 argmgesnpv tgntcdnvkq raalidclap drrveievkg ikdvvtqpqa
HAX8865859	Escherichia coli UTImcc302 human urine, France	PUBMED 30286803	VII	1 mkktaiaiav alagfatvaq aapkdntwyt gaklgwsqyh dtgfidnngp thenqlgaga<br>61 fggyqvnpyv gfemgydwlg rmpykgsven gaykaqgvql taklgypitd dldvytrlgg<br>121 mvwradtksn fdgknhdtgv spvfasgvey aitpeiatrl eyqwtnnigd ahtigtrpdn<br>181 gmlslgvsyr fgqgeaapvv apapapapev qtkhftlksd vlftfnkatl kpegqaaldq<br>241 lysqlsnldp kdgsvvvlgy tdrigsdayn qalserraqs vvdyliskgi padkisargm<br>301 gesnpvtgnt cdnvkqraal idclapdrrv eievkgikdv vtqpqa
EFH3094454	ECOL-19-VL-OH-WA-0011, Washington, urine (canis lupus)	n/a	I	1 mkktaiaiav alagfatvaq aapkdntwyt gaklgwsqyh dtgfinnngp thenqlgaga<br>61 fggyqvnpyv gfemgydwlg rmpykvsven gaykaqgvql taklgypitd dldvytrlgg<br>121 mvwradtksn vygknhdtgv spvfaggvey aitpeiatrl eyqwtnnigd ahtigtrpdn<br>181 gmlslgvsyr fgqgeaapvv apapapapev qtkhftlksd vlftfnkatl kpegqaaldq<br>241 lysqlsnldp kdgsvvvlgy tdrigsdayn qalserraqs vvdyliskgi padkisargm<br>301 gesnpvtgnt cdnvkqraal idclapdrrv eievkgikdv vtqpqa
AJB38777	Escherichia coli APEC IMT5155, chicken	n/a	I	1 mddneaqkmk ktaiaiaval agfatvaqaa pkdntwytga klgwsqyhdt gfinnngpth<br>61 enqlgagafg gyqvnpyvgf emgydwlgrm pykgsvenga ykaqgvqlta klgypitddl<br>121 dvytrlggmv wradtksnvy gknhdtgvsp vfaggveyai tpeiatrley qwtnnigdah<br>181 tigtrpdngm lslgvsyrfg qgeaapvvap apapapevqt khftlksdvl ftfnkatlkp<br>241 egqaaldqly sqlsnldpkd gsvvvlgytd rigsdaynqa lserraqsvv dyliskgipa<br>301 dkisargmge snpvtgntcd nvkqraalid clapdrrvei evkgikdvvt qpqa

Table S1: 100% identity matches to delta allele

EHD6720155	Salmonella enterica subsp. enterica serovar Enteritidis human stool, Canada, 2009	n/a	I	1 lgwsqyhdtg finnngpthe nqlgagafgg yqvnpyvgfe mgydwlgrmp ykgsvengay<br>61 kaqgvqltak lgypitddld vytrlggmvw radtksnvyg knhdtgvspv faggveyait<br>121 peiatrleyq wtnnigdaht igtrpdngml slgvsyrfgq geaapvvapa papapevqtk<br>181 hftlksdvlf tfnkatlkpe gqaaldqlys qlsnldpkdg svvvlgytdr igsdaynqal<br>241 serraqsvvd yliskgipad kisargmges npvtgntcdn vkqraalidc lapdrrveie<br>301 vkgikdvvtq pqa
EFC7752139	strain 382825, human isolate, United Kingdom	n/a	III	1 mkktaiaiav alagfatvaq aapkdntwyt gaklgwsqyh dtgfipnngp thenqlgaga<br>61 fggyqvnpyv gfemgydwlg rmpykgdni!n gaykaqgvql taklgypitd dldvytrlgg<br>121 mvwradtkan vpggasfkdh dtgvspvfag gveyaitpei atrleyqwtn nigdahtigt<br>181 rpdngmlslg vsyrfgqgea apvvapapap apevqtkhft lksdvlftfn katlkpegqa<br>241 aldqlysqls nldpkdgsvv vlgytdrigs daynqalser raqsvvdyli skgipadkis<br>301 argmgesnpv tgntcdnvkq raalidclap drrveievkg ikdvvtqpqa
EFF9255879	PNUSAE044635, USA	n/a	III	1 mkktaiaiav alagfatvaq aapkdntwyt gaklgwsqyh dtgfipnngp thenqlgaga<br>61 fggyqvnpyv gfemgydwlg rmpykgdni!n vaykaqgvql taklgypitd dldiytrlgg<br>121 mvwradtkan vpggasfkdh dtgvspvfag gveyaitpei atrleyqwtn nigdahtigt<br>181 rpdngmlslg vsyrfgqgea apvvapapap apevqtkhft lksdvlftfn katlkpegqa<br>241 aldqlysqls nldpkdgsvv vlgytdrigs daynqalser raqsvvdyli skgipadkis<br>301 argmgesnpv tgntcdnvkq raalidclap drrveievkg ikdvvtqpqa
HAN7992863	UTI_22, urine, Washington DC	n/a	III	1 mkktaiaiav alagfatvaq aapkdntwyt gaklgwsqyh dtgfipnngp thenqlgaga<br>61 fggyqvnpyv gfemgydwlg rmpykgdni!n gaykaqgvql taklgypitd dldiytrlgg<br>121 mvwradtkan vpggasfkdh dtgvspvfas gveyaitpei atrleyqwtn nigdahtigt<br>181 rpdngmlslg vsyrfgqgea apvvapapap apevqtkhft lksdvlftfn katlkpegqa<br>241 aldqlysqls nldpkdgsvv vlgytdrigs daynqalser raqsvvdyli skgipadkis<br>301 argmgesnpv tgntcdnvkq raalidclap drrveievkg ikdvvtqpqa
ASO87464	E. coli PAR, cockatoo feces, China	n/a	IV	1 mddneaqkmk ktaiaiaval agfatvaqaa pkdntwytga klgwsqyhdt gfinnngpth<br>61 enqlgagafg gyqvnpyvgf emgydwlgrm pykgsvenga ykaqgvqlta klgypitddl<br>121 dvytrlggmv wradtksnfd gknhdtgvsp vfaggveyai tpeiatrley qwtnnigdah<br>181 tigtrpdngm lslgvsyrfg qgeaapvvap apapapevqt khftlksdvl ftfnkatlkp<br>241 egqaaldqly sqlsnldpkd gsvvvlgytd rigsdaynqa lserraqsvv dyliskgipa<br>301 dkisargmge snpvtgntcd nvkqraalid clapdrrvei evkgikdvvt qpqa
EFB5273416	ECOL-19-VL-NY-MS-0012, urine from Canis lupus familiaris, USA MS (Mississippi? Missouri?), 2019	n/a	IV	1 mkktaiaiav alagfatvaq aapkdntwyt gaklgwslyh dtgfinnngp thenqlgaga<br>61 fggyqvnpyv gfemgydwlg rmpykgsven gaykaqgvql taklgypitd dldvytrlgg<br>121 mvwradtksn fdgknhdtgv spvfaggvey aitpeiatrl eyqwtnnigd ahtigtrpdn<br>181 gmlslgvsyr fgqgeaapvv apapapapev qtkhftlksd vlftfnkatl kpegqaaldq<br>241 lysqlsnldp kdgsvvvlgy tdrigsdayn qalserraqs vvdyliskgi padkisargm<br>301 gesnpvtgnt cdnvkqraal idclapdrrv eievkgikdv vtqpqa
EFN6810927	E. coli O110 PSU-1709, mouse, Pennsylvania, 2018	n/a	IV	1 mkktaiaiav alagfatvaq aapkdntwyt gaklgwsqyh dtgfinnngp thenqlgaga<br>61 fggyqvnpyv gfemgydwlg rmpykgsven gaykaqgvql taklgypitd dldvytrlgg<br>121 mvwradtksn fdgknhdtgv spvfaggvey aitpeiatrl eyqwtnnigd ahsigtrpdn<br>181 gmlslgvsyr fgqgeaapvv apapapapev qtkhftlksd vlftfnkatl kpegqaaldq<br>241 lysqlsnldp kdgsvvvlgy tdrigsdayn qalserraqs vvdyliskgi padkisargm<br>301 gesnpvtgnt cdnvkqraal idclapdrrv eievkgikdv vtqpqa
OSK51737	E. coli H588	n/a	IV	1 mkdltvlsrw ryswrilddn eaqkmkktai aiavalagfa tvaqaapkdn twytgaklgw<br>61 sqyhdtgfin nngpthenql gagafggyqv npyvgfemgy dwlgrmpykg svengaykaq<br>121 gvqltaklgy pitddldvyt rlggmvwrad tksnfdgknh dtgvspvfag gveyaitpei<br>181 atrleyqwtn nigdahtigt rpdngmlslg vsyrfgqgea apvvapapap apevqtkhft<br>241 lksdvlftfn katlkpegqa aldqlysqls nldpkdgsvv vlgytdrigs daynqalser<br>301 raqsvvdyli skgipadkis argmgesnpv tgntcdnvkq raalidclap drrveievkg<br>361 ikdvvtqpqa
EFO1582468	E. coli PSU-0851, Common Eider, Arkansas, 2015	n/a	other	1 mkktaiaiav alagfatvaq aapkdntwyt gaklgwsqyh dtgfinnngp thenqlgaga<br>61 fggyqvnpyv gfemgydwlg rmpykgieng aykaqgvqlt aklgypitdd ldvytrlggm<br>121 vwradtksnf dgknhdtgvs pvfaggveya itpeiatrle yqwtnnigda htigtrpdng<br>181 mlslgvsyrf gqgeaapvva papapapevq tkhftlksdv lftfnkatlk pegqaaldql<br>241 ysqlsnldpk dgsvvvlgyt drigsdaynq alserraqsv vdyliskgip adkisargmg<br>301 esnpvtgntc dnvkqraali dclapdrrve ievkgikdvv tqpqa
EHT7733901	PNUSAE076594, Chicken ceca, South Carolina, 2021	n/a	other	1 mkktaiaiav alagfatvaq aapkdntwyt gaklgwsqyh dtgfinnngp thenqlgaga<br>61 fggyqvnpyv gfemgydwlg rmpykgdni!n gaykaqgvql taklgypitd dldvytrlgg<br>121 mvwrvdtksn vygknhdtgv spvfaggvey aitpeiatrl eyqwtnnigd ahtigtrpdn<br>181 gmlslgvsyr fgqgeaapvv apapapapev qtkhftlksd vlftfnkatl kpegqaaldq<br>241 lysqlsnldp kdgsvvvlgy tdrigsdayn qalserraqs vvdyliskgi padkisargm<br>301 gesnpvtgnt cdnvkqraal idclapdrrv eievkgikdv vtqpqa
ETE12795	E. coli LAU-EC8, human gastrointestinal isolate, Lebanon, 2013	n/a	other	1 mlsrwryswr ilddneaqkm kktaiaiava lagfatvaqa apkdntwytg aklgwsqyhd<br>61 tgfinnngpt henqlgagaf ggyqvnpyvg femgydwlgr mpykgdni!ng aykaqgvqlt<br>121 aklgypitdd ldvytrlggm vwradtksnv ygknhdtgvs pvfaggveya itpeiatrle<br>181 yqwtnnigda htigtrpdng mlslgvsyrf gqgeaapvva papapapevq tkhftlksdv<br>241 lftfnkatlk pegqaaldql ysqlsnldpk dgsvvvlgyt drigsdaynq alserraqsv<br>301 vdyliskgip adkisargmg esnpvtgntc dnvkqraali dclapdrrve ievkgikdvv<br>361 tqpqa
QKN47346	SCU-120, rectal swab from healthy college student, USA 2016	n/a	other	1 maiavalagf atvaqaapkd ntwytgaklg wsqyhdtgfi nnngpthenq lgagafggyq<br>61 vnpyvgfemg ydwlgrmpyk gdningayka qgvqltaklg ypitddldvy trlggmvwra<br>121 dtksnvygkn hdtgvspvfa ggveyaitpe iatrleyqwt nnigdahtig trpdngmlsl<br>181 gvsyrfgqge aapvvapapa papevqtkhf tlksdvlftf nkatlkpegq aaldqlysql<br>241 snldpkdgsv vvlgytdrig sdaynqalse rraqsvvdyl iskgipadki sargmgesnp<br>301 vtgntcdnvk qraalidcla pdrrveievk gikdvvtqpq a

Table S1: 100% identity matches to delta allele

EGF1723824	PNUSAE059262, human stool, Washington State (2019)	n/a	other	1 mkktaiaiav alagfatvaq aapkdntwyt gaklgwsqyh dtgfipnngp thenqlgaga<br>61 fggyqvnpyv gfemgydwlg rmpykgdni!n gaykaqgvql taklgypitd dldiytrlgg<br>121 mvwradtkan ipggasfkdh dtgvspvfag gveyaitpei atrleyqwtn nigdahtigt<br>181 rpdngmlslg vsyrfgqgea apvvapapap apevqtkhft lksdvlftfn katlkpegqa<br>241 aldqlysqls nldpkdgsvv vlgytdrigs daynqalser raqsvvdyli skgipadkis<br>301 argmgesnpv tgntcdnvkq raalidclap drrveievkg ikdvvtqpqa
MBB7653637	88.0597, primate, 1988 (year), Alabma	n/a	other	1 mkktaiaiav alagfatvaq aapkdntwyt gaklgwsqyh dtgfipnngp thenqlgaga<br>61 fggyqvnpyv gfemgydwlg rmpykgdni!n gaykaqgvql taklgypitd dldiytrlgg<br>121 mvwradtkan vpggasfknh dtgvspvfag gveyaitpei atrleyqwtn nigdahtigt<br>181 rpdngmlslg vsyrfgqgea apvvapapap apevqtkhft lksdvlftfn katlkpegqa<br>241 aldqlysqls nldpkdgsvv vlgytdrigs daynqalser raqsvvdyli skgipadkis<br>301 argmgesnpv tgntcdnvkq raalidclap drrveievkg ikdvvtqpqa
EEC8143277	human isolate 441436, UK, 2017	n/a	other	1 mkktaiaiav alagfatvaq aapkdntwyt gaklgwsqyh dtgfipnngp thenqlgaga<br>61 fggyqvnpyv gfemgydwlg rmpykgdni!n gaykaqgvql taklgypitd dldvytrlgg<br>121 mvwradtksn vpggastkdh dtgvspvfag gveyaitpei atrleyqwtn nigdahtidt<br>181 rpdngmlslg vsyrfgqgea apvvapapap apevqtkhft lksdvlftfn katlkpegqa<br>241 aldqlysqls nldpkdgsvv vlgytdrigs daynqalser raqsvvdyli skgipadkis<br>301 argmgesnpv tgntcdnvkq raalidclap drrveievkg ikdvvtqpqa
EGR8448086	human isolate 1041391, UK, 2020	n/a	other	1 mkktaiaiav alagfatvaq aapkdntwyt gaklgwsqyh dtgfipnngp thenqlgaga<br>61 fggyqvnpyv gfemgydwlg rmpykgdni!n gaykaqgvql taklgypitd dldvytrlgg<br>121 mvwradsksn vpggastkdh dtgvspvfag gveyaitpei atrleyqwtn nigdahtigt<br>181 rpdngmlslg vsyrfgqgea apvvapapap apevqtkhft lksdvlftfn katlkpegqa<br>241 aldqlysqls nldpkdgsvv vlgytdrigs daynqalser raqsvvdyli skgipadkis<br>301 argmgesnpv tgntcdnvkq raalidclap drrveievkg ikdvvtqpqa
MBI9970753	EC00657, human urine, University of Pittsburgh Medical Center	n/a	other	1 mkktaiaiav alagfatvaq aapkdntwyt gaklgwsqyh dtgfipnngp thenqlgaga<br>61 fggyqvnpyv gfemgydwlg rmpykgdni!n gaykaqgvql taklgypitd dldiytrlgg<br>121 mvwradtkan epggasfkdh dtgvspvfag gveyaitpei atrleyqwtn nigdahtigt<br>181 rpdngmlslg vsyrfgqgea apvvapapap apevqtkhft lksdvlftfn katlkpegqa<br>241 aldqlysqls nldpkdgsvv vlgytdrigs daynqalser raqsvvdyli skgipadkis<br>301 argmgesnpv tgntcdnvkq raalidclap drrveievkg ikdvvtqpqa
EFM53798	NC101	n/a	VII	1 mlsrwryswr ilddneaqkm kktaiaiava lagfatvaqa apkdntwytg aklgwsqyhd<br>61 tgfidnngpt henqlgagaf ggyqvnpyvg femgydwlgr mpykgsveng aykaqgvqlt<br>121 aklgypitdd ldvytrlggm vwradtksnf dgknhdtgvs pvfaggveya itpeiatrle<br>181 yqwtnnigda htigtrpdng mlslgvsyrf gqgeaapvva papapapevq tkhftlksdv<br>241 lftfnkatlk pegqaaldql ysqlsnldpk dgsvvvlgyt drigsdaynq alserraqsv<br>301 vdyliskgip adkisargmg esnpvtgntc dnvkqraali dclapdrrve ievkgikdvv<br>361 tqpqa
EHO5965289	PNUSAE073054	n/a	VII	1 mkktaiaiav alagfatvaq aapkdntwyt gaklgwsqyh digfidnngp thenqlgaga<br>61 fggyqvnpyv gfemgydwlg rmpykgsven gaykaqgvql taklgypitd dldvytrlgg<br>121 mvwradtksn fdgknhdtgv spvfaggvey aitpeiatrl eyqwtnnigd ahtigtrpdn<br>181 gmlslgvsyr fgqgeaapvv apapapapev qtkhftlksd vlftfnkatl kpegqaaldq<br>241 lysqlsnldp kdgsvvvlgy tdrigsdayn qalserraqs vvdyliskgi padkisargm<br>301 gesnpvtgnt cdnvkqraal idclapdrrv eievkgikdv vtqpqa
KKA62344	E. coli 9.1649, pig isolate	n/a	VII	1 mddneaqkmk ktaiaiaval agfatvaqaa pkdntwytga klgwsqyhdt gfidnngpth<br>61 enqlgagafg gyqvnpyvgf emgydwlgrm pykgsvenga ykaqgvqlta klgypitddl<br>121 dvytrlggmv wradtksnfd gknhdtgvsp vfaggveyai tpeiatrley qwtnnigdah<br>181 tigtrpdngm lslgvsyrfg qgeaapvvap apapapevqt khftlksdvl ftfnkatlkp<br>241 egqaaldqly sqlsnldpkd gsvvvlgytd rigsdaynqa lserraqsvv dyliskgipa<br>301 dkisargmge snpvtgntcd nvkqraalid clapdrrvei evkgikdvvt qpqa
EHF8087547	E. coli strain 1125498, human, United Kingdom, 2021		I	1 mkktaiaiav alagfatvaq aapkdntwyt gaklgwsqyh dtgfinnngp thenqlgaga<br>61 fggyqvnpyv gfemgydwlg rmpykgsven daykaqgvql taklgypitd dldvytrlgg<br>121 mvwradtksn vygknhdtgv spvfaggvey aitpeiatrl eyqwtnnigd ahtigtrpdn<br>181 gmlslgvsyr fgqgeaapvv apapapapev qtkhftlksd vlftfnkatl kpegqaaldq<br>241 lysqlsnldp kdgsvvvlgy tdrigsdayn qalserraqs vvdyliskgi padkisargm<br>301 gesnpvtgnt cdnvkqraal idclapdrrv eievkgikdv vtqpqa
EGI1169706	ECOL-19-VL-OH-WA-0027, Urine (Canis lupus familiaris), Washington State, 2019		IV	1 mkktaiaiav alagfatvaq aapkdntwyt gaklgwsqyh dtgfinnngp thenqlgaga<br>61 fggyqvnpyv gfemgydwlg rmpykgsven gaykaqgvql taklgypitd dldvytrlgg<br>121 mvwradtksn fdgknhdtgv spvfaggvey aitpeiatrl eyqwtnnigd ahtigtrldn<br>181 gmlslgvsyr fgqgeaapvv apapapapev qtkhftlksd vlftfnkatl kpegqaaldq<br>241 lysqlsnldp kdgsvvvlgy tdrigsdayn qalserraqs vvdyliskgi padkisargm<br>301 gesnpvtgnt cdnvkqraal idclapdrrv eievkgikdv vtqpqa
HAL1663844	E. coli G167 ST404, human, Washington state, 2010		IV	1 mkktaiaiav alagfatvaq aapkdntwyt gaklgwsqyh dtgfinnngp thenqlgaga<br>61 fggyqvnpyv gfemgydwlg rmpykgsven gaykaqgvql taklgypiad dldvytrlgg<br>121 mvwradtksn fdgknhdtgv spvfaggvey aitpeiatrl eyqwtnnigd ahtigtrpdn<br>181 gmlslgvsyr fgqgeaapvv apapapapev qtkhftlksd vlftfnkatl kpegqaaldq<br>241 lysqlsnldp kdgsvvvlgy tdrigsdayn qalserraqs vvdyliskgi padkisargm<br>301 gesnpvtgnt cdnvkqraal idclapdrrv eievkgikdv vtqpqa
MBW2916868	Escherichia coli SCPM-O--8971, human urine, Russia, 2016			1 tgvspvfagg veyaitpeia trleyqwtnn igdahtigtr pdngmlslgv syrfgqgeaa<br>61 pvvapapapa pevqtkhftl ksdvlftfnk atlkpegqaa ldqlysqlsn ldpkdgsvvv<br>121 lgytdrigsd aynqalserr aqsvvdylis kgipadkisa rgmgesnpvt gntcdnvkqr<br>181 aalidclapd rrveievkgi kdvvtqpqa
PNE00854	Escherichia coli SP-16 Combo Netherlands:Amsterdam 1997			1 hdtgvspvfa ggveyaitpe iatrleyqwt nnigdahtig trpdngmlsl gvsyrfgqge<br>61 aapvvapapa papevqtkhf tlksdvlftf nkatlkpegq aaldqlysql snldpkdgsv<br>121 vvlgytdrig sdaynqalse rraqsvvdyl iskgipadki sargmgesnp vtgntcdnvk<br>181 qraalidcla pdrrveievk gikdvvtqpq a

Table S1: 100% identity matches to delta allele

RIC98197	E. coli S339 O1:K1, cerebrospinal fluid, human, France, 2007			1 ldvytrlggm vwradtksnv ygknhdtgvs pvfaggveya itpeiatrle yqwtnnigda<br>61 htigtrpdng mlslgvsyrf gqgeaapvva papapapevq tkhftlksdv lftfnkatlk<br>121 pegqaaldql ysqlsnldpk dgsvvvlgyt drigsdaynq alserraqsv vdyliskgip<br>181 adkisargmg esnpvtgntc dnvkqraali dclapdrrve ievkgikdvv tqpqa
MBF9178793	E. coli E3, human blood, South Africa, 2018			1 trpdngmlsl gvsyrfgqge aapvvapapa papevqtkhf tlksdvlftf nkatlkpegq<br>61 aaldqlysql snldpkdgsv vvlgytdrig sdaynqalse rraqsvvdyl iskgipadki<br>121 sargmgesnp vtgntcdnvk qraalidcla pdrrveievk gikdvvtqpq a
