# Supplementary material for: Allelic variation of Escherichia coli outer membrane protein A: Impact on cell surface properties, stress tolerance and allele distribution
Source: PLoS One. 2022 Oct 13;17(10):e0276046. doi: 10.1371/journal.pone.0276046 (PMC9560509; doi:10.1371/journal.pone.0276046)
Supplement: S1 File — (DOCX) [file pone.0276046.s003.docx]

>I, alpha

MKKTAIAIAVALAGFATVAQAAPKDNTWYTGAKLGWSQYHDTGFI**N**NNGPTHENQLGAGAFGGYQVNPYVGFEMGYDWLGRMPYKG**SVE**NGAYKAQGVQLTAKLGYPITDDLD**I**YTRLGGMVWRADTK**SNVYG**K**N**HDTGVSPVFAGGVE**Y**AITPEIATRLEYQWTNNIGDA**H**TIGTRPDNG**M**LSLGVSYRFGQGE**A**APVV**A**PAPAPAPEVQTKHFTLKSDVLF**N**FNKATLKPEGQAALDQLYSQLSNLDPKDGSVVVLGYTDRIGSDAYNQ**G**LSERRAQSVVDYLISKGIP**A**DKISARGMGESNPVTGNTCDNVKQRAALIDCLAPDRRVEIEVKGIKDVVTQPQA

>II, alpha

MKKTAIAIAVALAGFATVAQAAPKDNTWYTGAKLGWSQYHDTGFI**P**NNGPTHENQLGAGAFGGYQVNPYVGFEMGYDWLGRMPYKG**DNI**NGAYKAQGVQLTAKLGYPITDDLD**V**YTRLGGMVWRADTK**ANVPGGASF**K**D**HDTGVSPVFAGGVE**Y**AITPEIATRLEYQWTNNIGDA**N**TIGTRPDNG**L**LSLGVSYRFGQGE**A**APVV**A**PAPAPAPEVQTKHFTLKSDVLF**N**FNKATLKPEGQAALDQLYSQLSNLDPKDGSVVVLGYTDRIGSDAYNQ**G**LSERRAQSVVDYLISKGIP**A**DKISARGMGESNPVTGNTCDNVKQRAALIDCLAPDRRVEIEVKGIKDVVTQPQA

>III, gamma

MKKTAIAIAVALAGFATVAQAAPKDNTWYTGAKLGWSQYHDTGFI**P**NNGPTHENQLGAGAFGGYQVNPYVGFEMGYDWLGRMPYKG**DNI**NGAYKAQGVQLTAKLGYPITDDLD**I**YTRLGGMVWRADTK**ANVPGGASF**K**D**HDTGVSPVFAGGVE**Y**AITPEIATRLEYQWTNNIGDA**H**TIGTRPDNG**M**LSLGVSYRFGQGE**V**APVV**A**PAPAPAPEVQTKHFTLKSDVLF**T**FNKATLKPEGQAALDQLYSQLSNLDPKDGSVVVLGYTDRIGSDAYNQ**A**LSERRAQSVVDYLISKGIP**A**DKISARGMGESNPVTGNTCDNVKQRAALIDCLAPDRRVEIEVKGIKDVVTQPQA

>IV, beta

MKKTAIAIAVALAGFATVAQAAPKDNTWYTGAKLGWSQYHDTGFI**N**NNGPTHENQLGAGAFGGYQVNPYVGFEMGYDWLGRMPYKG**SVE**NGAYKAQGVQLTAKLGYPITDDLD**V**YTRLGGMVWRADTK**SNFDG**K**N**HDTGVSPVFAGGVE**Y**AITPEIATRLEYQWTNNIGDA**H**TIGTRPDNG**M**LSLGVSYRFGQGE**A**APVV**A**PAPAPAPEVQTKHFTLKSDVLF**T**FNKATLKPEGQAALDQLYSQLSNLDPKDGSVVVLGYTDRIGSDAYNQ**A**LSERRAQSVVDYLISKGIP**A**DKISARGMGESNPVTGNTCDNVKQRAALIDCLAPDRRVEIEVKGIKDVVTQPQA

>V, alpha

MKKTAIAIAVALAGFATVAQAAPKDNTWYTGAKLGWSQYHDTGFI**D**NNGPTHENQLGAGAFGGYQVNPYVGFEMGYDWLGRMPYKG**SVE**NGAYKAQGVQLTAKLGYPITDDLD**V**YTRLGGMVWRADTK**AHNNVTGESE**K**N**HDTGVSPVFAGGVE**W**AITPEIATRLEYQWTNNIGDA**H**TIGTRPDNG**L**LSLGVSYRFGQGE**A**APVV**A**PAPAPAPEVQTKHFTLKSDVLF**N**FNKATLKPEGQAALDQLYSQLSNLDPKDGSVVVLGYTDRIGSDAYNQ**G**LSERRAQSVVDYLISKGIP**A**DKISARGMGESNPVTGNTCDNVKQRAALIDCLAPDRRVEIEVKGIKDVVTQPQA

>VI, alpha

MKKTAIAIAVALAGFATVAQAAPKDNTWYTGAKLGWSQYHDTGFI**D**NNGPTHENQLGAGAFGGYQVNPYVGFEMGYDWLGRMPYKG**DNI**NGAYKAQGVQLTAKLGYPITDDLD**V**YTRLGGMVWRADTK**SNVYG**K**N**HDTGVSPVFAGGVE**Y**AITPEIATRLEYQWTNNIGDA**H**TIGTRPDNG**L**LSLGVSYRFGQGE**A**APVV**A**PAPAPAPEVQTKHFTLKSDVLF**N**FNKATLKPEGQAALDQLYSQLSNLDPKDGSVVVLGYTDRIGSDAYNQ**G**LSERRAQSVVDYLISKGIP**A**DKISARGMGESNPVTGNTCDNVKQRAALIDCLAPDRRVEIEVKGIKDVVTQPQA

>VII, beta

MKKTAIAIAVALAGFATVAQAAPKDNTWYTGAKLGWSQYHDTGFI**D**NNGPTHENQLGAGAFGGYQVNPYVGFEMGYDWLGRMPYKG**SVE**NGAYKAQGVQLTAKLGYPITDDLD**V**YTRLGGMVWRADTK**SNFDG**K**N**HDTGVSPVFAGGVE**Y**AITPEIATRLEYQWTNNIGDA**H**TIGTRPDNG**M**LSLGVSYRFGQGE**A**APVV**A**PAPAPAPEVQTKHFTLKSDVLF**T**FNKATLKPEGQAALDQLYSQLSNLDPKDGSVVVLGYTDRIGSDAYNQ**G**LSERRAQSVVDYLISKGIP**A**DKISARGMGESNPVTGNTCDNVKQRAALIDCLAPDRRVEIEVKGIKDVVTQPQA

>EHEC ATCC 35150 (V, alpha)
MKKTAIAIAVALAGFATVAQAAPKDNTWYTGAKLGWSQYHDTGFI**D**NNGPTHENQLGAGAFGGYQVNPYVGFEMGYDWLGRMPYKG**SVE**NGAYKAQGVQLTAKLGYPITDDLDVYTRLGGMVWRADTK**AHNNVTGESE**KNHDTGVSPVFAGGVEWAITPEIATRLEYQWTNNIGDA**H**TIGTRPDNGLLSLGVSYRFGQGEAAPVV**A**PAPAPAPEVQTKHFTLKSDVLF**N**FNKATLKPEGQAALDQLYSQLSNLDPKDGSVVVLGYTDRIGSDAYNQ**G**LSERRAQSVVDYLISKGIP**A**DKISARGMGESNPVTGNTCDNVKQRAALIDCLAPDRRVEIEVKGIKDVVTQPQA

>EPEC O83:H1 str. NRG 857C (IV, delta)
MKKTAIAIAVALAGFATVAQAAPKDNTWYTGAKLGWSQYHDTGFI**N**NNGPTHENQLGAGAFGGYQVNPYVGFEMGYDWLGRMPYKG**SVE**NGAYKAQGVQLTAKLGYPITDDLDVYTRLGGMVWRADTK**SNFDG**KNHDTGVSPVFAGGVEYAITPEIATRLEYQWTNNIGDA**H**TIGTRPDNGMLSLGVSYRFGQGEAAPVV**A**PAPAPAPEVQTKHFTLKSDVLF**T**FNKATLKPEGQAALDQLYSQLSNLDPKDGSVVVLGYTDRIGSDAYNQ**A**LSERRAQSVVDYLISKGIP**A**DKISARGMGESNPVTGNTCDNVKQRAALIDCLAPDRRVEIEVKGIKDVVTQPQA

>ETEC str. E24377A (II, alpha)
MKKTAIAIAVALAGFATVAQAAPKDNTWYTGAKLGWSQYHDTGFI**P**NNGPTHENQLGAGAFGGYQVNPYVGFEMGYDWLGRMPYKG**DNI**NGAYKAQGVQLTAKLGYPITDDLDVYTRLGGMVWRADTK**ANVPGGASF**KDHDTGVSPVFAGGVEYAITPEIATRLEYQWTNNIGDA**N**TIGTRPDNGLLSLGVSYRFGQGEAAPVV**A**PAPAPAPEVQTKHFTLKSDVLF**N**FNKATLKPEGQAALDQLYSQLSNLDPKDGSVVVLGYTDRIGSDAYNQ**G**LSERRAQSVVDYLISKGIP**A**DKISARGMGESNPVTGNTCDNVKQRAALIDCLAPDRRVEIEVKGIKDVVTQPQA

>*E. coli* str. ECONIH1 (other, delta)
MKKTAIAIAVALAGFATVAQAAPKDNTWYTGAKLGWSQYHDTGFI**N**NNGPTHENQLGAGAFGGYQVNPYVGFEMGYDWLGRMPYKG**DNI**NGAYKAQGVQLTAKLGYPITDDLDVYTRLGGMVWRADTK**SNVYG**KNHDTGVSPVFAGGVEYAITPEIATRLEYQWTNNIGDA**H**TIGTRPDNGMLSLGVSYRFGQGEAAPVV**A**PAPAPAPEVQTKHFTLKSDVLF**T**FNKATLKPEGQAALDQLYSQLSNLDPKDGSVVVLGYTDRIGSDAYNQ**A**LSERRAQSVVDYLISKGIP**A**DKISARGMGESNPVTGNTCDNVKQRAALIDCLAPDRRVEIEVKGIKDVVTQPQA

>EHEC str. LB226692 O104:H4 (I, alpha)
MKKTAIAIAVALAGFATVAQAAPKDNTWYTGAKLGWSQYHDTGFI**N**NNGPTHENQLGAGAFGGYQVNPYVGFEMGYDWLGRMPYKG**SVE**NGAYKAQGVQLTAKLGYPITDDLDIYTRLGGMVWRADTK**SNVYG**KNHDTGVSPVFAGGVEYAITPEIATRLEYQWTNNIGDA**H**TIGTRPDNGMLSLGVSYRFGQGEAAPVV**A**PAPAPAPEVQTKHFTLKSDVLF**N**FNKATLKPEGQAALDQLYSQLSNLDPKDGSVVVLGYTDRIGSDAYNQ**G**LSERRAQSVVDYLISKGIP**A**DKISARGMGESNPVTGNTCDNVKQRAALIDCLAPDRRVEIEVKGIKDVVTQPQA

>EAEC str 042 (other, beta)

MKKTAIAIAVALAGFATVAQAAPKDNTWYTGAKLGWSQYHDTGFI**D**NNGPTHENQLGAGAFGGYQVNPYVGFEMGYDWLGRMPYKG**DNI**NGAYKAQGVQLTAKLGYPITDDLDVYTRLGGMVWRADTK**SNVPGGVST**KDHDTGVSPVFAGGVEYAITPEIATRLEYQWTNNIGDA**H**TIGTRPDNGMLSLGVSYRFGQGEAAPVV**A**PAPAPAPEVQTKHFTLKSDVLF**T**FNKATLKPEGQAALDQLYSQLSNLDPKDGSVVVLGYTDRIGSDAYNQ**G**LSERRAQSVVDYLISKGIP**A**DKISARGMGESNPVTGNTCDNVKQRAALIDCLAPDRRVEIEVKGIKDVVTQPQA

>ETEC str H10407 (I, alpha)

MKKTAIAIAVALAGFATVAQATPKDNTWYTGAKLGWSQYHDTGFI**N**NNGPTHENQLGAGAFGGYQVNPYVGFEMGYDWLGRMPYKG**SVE**NGAYKAQGVQLTAKLGYPITDDLDIYTRLGGMVWRADTK**SNVYG**KNHDTGVSPVFAGGVEYAITPEIATRLEYQWTNNIGDA**H**TIGTRPDNGMLSLGVSYRFGQGEAAPVV**A**PAPAPAPEVQTKHFTLKSDVLF**N**FNKATLKPEGQAALDQLYSQLSNLDPKDGSVVVLGYTDRIGSDAYNQ**G**LSERRAQSVVDYLISKGIP**A**DKISARGMGESNPVTGNTCDNVKQRAALIDCLAPDRRVEIEVKGIKDVVTQPQA

>STEC str 2011EL-1675A O104:H4 (I, alpha)

MKKTAIAIAVALAGFATVAQAAPKDNTWYTGAKLGWSQYHDTGFI**N**NNGPTHENQLGAGAFGGYQVNPYVGFEMGYDWLGRMPYKG**SVE**NGAYKAQGVQLTAKLGYPITDDLDIYTRLGGMVWRADTK**SNVYG**KNHDTGVSPVFAGGVEYAITPEIATRLEYQWTNNIGDA**H**TIGTRPDNGMLSLGVSYRFGQGEAAPVV**A**PAPAPAPEVQTKHFTLKSDVLF**N**FNKATLKPEGQAALDQLYSQLSNLDPKDGSVVVLGYTDRIGSDAYNQ**G**LSERRAQSVVDYLISKGIP**A**DKISARGMGESNPVTGNTCDNVKQRAALIDCLAPDRRVEIEVKGIKDVVTQPQA

>STEC str RM12581 O145:H28 (V, alpha)

MKKTAIAIAVALAGFATVAQAAPKDNTWYTGAKLGWSQYHDTGFI**D**NNGPTHENQLGAGAFGGYQVNPYVGFEMGYDWLGRMPYKG**SVE**NGAYKAQGVQLTAKLGYPITDDLDVYTRLGGMVWRADTK**AHNNVTGESE**KNHDTGVSPVFAGGVEWAITPEIATRLEYQWTNNIGDA**H**TIGTRPDNGLLSLGVSYRFGQGEAAPVV**A**PAPAPAPEVQTKHFTLKSDVLF**N**FNKATLKPEGQAALDQLYSQLSNLDPKDGSVVVLGYTDRIGSDAYNQ**G**LSERRAQSVVDYLISKGIP**A**DKISARGMGESNPVTGNTCDNVKQRAALIDCLAPDRRVEIEVKGIKDVVTQPQA

>STEC str 2010C-3508 O145:NM (V, alpha)

MKKTAIAIAVALAGFATVAQAAPKDNTWYTGAKLGWSQYHDTGFI**D**NNGPTHENQLGAGAFGGYQVNPYVGFEMGYDWLGRMPYKG**SVE**NGAYKAQGVQLTAKLGYPITDDLDVYTRLGGMVWRADTK**AHNNVTGESE**KNHDTGVSPVFAGGVEWAITPEIATRLEYQWTNNIGDA**H**TIGTRPDNGLLSLGVSYRFGQGEAAPVV**A**PAPAPAPEVQTKHFTLKSDVLF**N**FNKATLKPEGQAALDQLYSQLSNLDPKDGSVVVLGYTDRIGSDAYNQ**G**LSERRAQSVVDYLISKGIP**A**DKISARGMGESNPVTGNTCDNVKQRAALIDCLAPDRRVEIEVKGIKDVVTQPQA

>STEC *E. coli* O157:H7 str 1125 (I, other)

MKKTAIAIAVALAGFATVAQAAPKDNTWYTGAKLGWSQYHDTGFI**N**NNGPTHENQLGAGAFGGYQVNPYVGFEMGYDWLGRMPYKG**SVE**NGAYKAQGVQLTAKLGYPITDDLDIYTRLGGMVWRADTK**SNVYG**KNHDTGVSPVFAGGVEYAITPEIATRLEYQWTNNIGDA**H**TIGTRPDNGMLSLGVSYRFGQGEAAPVV**A**PAPAPAPEVQTKHFTLKSDVLF**N**FNKATLKPEGQAALDQLYSQ**L**SNLDPKDGSVVVLGYTDRIGSDAYNQGLSERRAQSVVDYLISKGIP**A**DKISARGMGESNPVTGNTCDNVKQRAALIDCLAPDRRVEIEVKGIKDVVTQPQA

>EIEC str 53638 gcontig_1105238512145 (I, alpha)

MKKTAIAIAVALAGFATVAQAAPKDNTWYTGAKLGWSQYHDTGFI**N**NNGPTHENQLGAGAFGGYQVNPYVGFEMGYDWLGRMPYKG**SVE**NGAYKAQGVQLTAKLGYPITDDLDIYTRLGGMVWRADTK**SNVYG**KNHDTGVSPVFAGGVEYAITPEIATRLEYQWTNNIGDA**H**TIGTRPDNGMLSLGVSYRFGQGEAAPVV**A**PAPAPAPEVQTKHFTLKSDVLF**N**FNKATLKPEGQAALDQLYSQLSNLDPKDGSVVVLGYTDRIGSDAYNQ**G**LSERRAQSVVDYLISKGIP**A**DKISARGMGESNPVTGNTCDNVKQRAALIDCLAPDRRVEIEVKGIKDVVTQPQA

>*E. albertii* str KF1 (other, alpha)

MKKTAIAIAVALAGFATVAQAAPKDNTWYTGAKLGWSQFHDTGFI**D**NNGPTHENQLGAGAFGGYQVNPYVGFEMGYDWLGRMPYKG**DNI**NGAYKAQGVQLTAKLGYPITDDLDVYTRLGGMVWRADTK**ANVPGGASF**KDHDTGVSPVFAGGVEYAITPEIATRLEYQWTNNIGDA**H**TIGTRPDNGLLSLGVSYRFGQGEAAPVV**A**PAPAPAPEVQTKHFTLKSDVLF**N**FNKATLKPEGQAALDQLYSQLSNLDPKDGSVVVLGYTDRIGSDAYNQ**G**LSERRAQSVVDYLISKGIP**S**DKISARGMGESNPVTGNTCDNVKQRAALIDCLAPDRRVEIEVKGIKDVVTQPQA

>*E. fergusonii* ATCC 35469 (other, alpha)
MKKTAIAIAVALAGFATVAQAAPKDNTWYTGAKLGWSQYHDTGFI**D**NNGPTHENQLGAGAFGGYQVNPYVGFEMGYDWLGRMPYKG**SVE**NGAYKAQGVQLTAKLGYPITDDLDIYTRLGGMVWRADTK**AHNNVTGESE**KNHDTGVSPVFAGGVEWAITPEIATRLEYQWTNNIGDA**N**TIGTRPDNGLLSLGVSYRFGQGEAAPVV**A**PAPAPAPEVQTKHFTLKSDVLF**N**FNKATLKPEGQAALDQLYSQLSNLDPKDGSVVVLGYTDRIGSDAYNQ**G**LSERRAQSVVDYLISKGIP**A**DKISARGMGESNPVTGNTCDNVKQRAALIDCLAPDRRVEIEVKGIKDVVTQPQA

>*E. vulneris* str. NBRC 102420 (other, alpha)
MKKTAIAIAVALAGFATVAQAAPKDNTWYTGAKLGWSQYHDNGFI**P**NDGPTREDQLGAGAYGGYQVNPYVGFELGYDWLGREPYKG**DNV**NGAFKSQGVQLTAKLGYPITDDVDIYTRLGGMVWRADAK**AQVPGTGASF**KDHDTGVSPVFAGGVEWAMTRDIATRLEYQWVNNIGDA**K**TLGTRQDNGMLSVGVSYRFGQQEDVAPVV**A**PAPAPAPQVSTKHFTLKSDVLF**N**FNKATLKPEGQQALDQMYSQLSNLDPKDGSVVVLGFTDRIGSDAYNQ**G**LSEKRAQSVVDYLISKGIP**S**NKISARGMGESNPVTGNTCDNVKARPALIDCLAPDRRVEIEVKGVKDVVTQPQA

>*S. enterica* str. FORC_015 (other, alpha)
MKKTAIAIAVALAGFATVAQAAPKDNTWYAGAKLGWSQYHDTGFI**N**NDGPTHENQLGAGAFGGYQVNPYVGFEMGYDWLGRMPYKG**DNI**NGAYKAQGVQLTAKLGYPITDDLDVYTRLGGMVWRADTK**SNVPGGPST**KDHDTGVSPVFAGGIEYAITPEIATRLEYQWTNNIGDA**N**TIGTRPDNGLLSVGVSYRFGQQEAAPVV**A**PAPAPAPEVQTKHFTLKSDVLF**N**FNKSTLKPEGQQALDQLYSQLSNLDPKDGSVVVLGFTDRIGSDAYNQ**G**LSEKRAQSVVDYLISKGIP**S**DKISARGMGESNPVTGNTCDNVKPRAALIDCLAPDRRVEIEVKGVKDVVTQPQA

>*S. dysenteriae* str. 1617 (V, alpha)

MKKTAIAITVALAGFATVAQAAPKDNTWYTGAKLGWSQYHDTGFI**D**NNGPTHENQLGAGAFGGYQVNPYVGFEMGYDWLGRMPYKG**SVE**NGAYKAQGVQLTAKLGYPITDDLDVYTRLGGMVWRADTK**AHNNVTGESE**KNHDTGVSPVFAGGVEWAITPEIATRLEYQWTNNIGDA**H**TIGTRPDNGLLSLGVSYRFGQGEAAPVV**A**PAPAPAPEVQTKHFTLKSDVLF**N**FNKATLKPEGQAALDQLYSQLSNLDPKDGSVVVLGYTDRIGSDAYNQ**G**LSERRAQSVVDYLISKGIP**A**DKISARGMGESNPVTGNTCDNVKQRAALIDCLAPDRRVEIEVKGIKDVVTQPQA

>*S. dysenteriae* str. 225-75 (I, alpha)

MKKTAIAIAVALAGFATVAQAAPKDNTWYTGAKLGWSQYHDTGFI**N**NNGPTHENQLGAGAFGGYQVNPYVGFEMGYDWLGRMPYKG**SVE**NGAYKAQGVQLTAKLGYPITDGLDIYTRLGGMVWRADTK**SNVYG**KNHDTGVSPVFAGGVEYAITPEIATRLEYQWTNNIGDA**H**TIGTRPDNGMLSLGVSYRFGQGEAAPVV**A**PAPAPAPEVQTKHFTLKSDVLF**N**FNKATLKPEGQAALDQLYSQLSNLDPKDGSVVVLGYTDRIGSDAYNQ**G**LSERRAQSVVDYLISKGIP**A**DKISARGMGESNPVTGNTCDNVKQRAALIDCLAPDRRVEIEVKGIKDVVTQPQA

>*S. dysenteriae* str. Sd197 (other, alpha)

MKKTAIAITVALAGFATVAQAAPKDNTWYTGAKLGWSQYHDTGFI**D**NNGPTHENQLGAGAFGGYQVNPYVGFEMGYDWLGRMPYKG**SVE**NGAYKAQGVQLTAKLGYPITDDLDIYTRLGGMVWRADTK**ANVPGGASF**KDHDTGVSPVFAGGVEYAITPEIATRLEYQWTNNIGDA**H**TIGTRPDNGLLSLGVSYRFGQGEAAPVV**A**PAPAPAPEVQTKHFTLKSDVLF**N**FNKATLKPEGQAALDQLYSQLSNLDPKDGSVVVLGYTDRIGSDAYNQ**G**LSERRAQSVVDYLISKGIP**A**DKISARGMGESNPVTGNTCDNVKQRAALIDCLAPDRRVEIEVKGIKDVVTQPQA

>*S. sonnei* str. 3226-85 (I, alpha)

MKKTAIAIAVALAGFATVAQAAPKDNTWYTGAKLGWSQYHDTGFI**N**NNGPTHENQLGAGAFGGYQVNPYVGFEMGYDWLGRMPYKG**SVE**NGAYKAQGVQLTAKLGYPITDDLDIYTRLGGMVWRADTK**SNVYG**KNHDTGVSPVFAGGVEYAITPEIATRLEYQWTNNIGDA**H**TIGTRPDNGMLSLGVSYRFGQGEAAPVV**A**PAPAPAPEVQTKHFTLKSDVLF**N**FNKATLKPEGQAALDQLYSQLSNLDPKDGSVVVLGYTDRIGSDAYNQ**G**LSERRAQSVVDYLISKGIP**A**DKISARGMGESNPVTGNTCDNVKQRAALIDCLAPDRRVEIEVKGIKDVVTQPQA

>*S. boydii* str. BS512 (I, alpha)

MKKTAIAIAVALAGFATVAQAAPKDNTWYTGAKLGWSQYHDTGFI**N**NNGPTHENQLGAGAFGGYQVNPYVGFEMGYDWLGRMPYKG**SVE**NGAYKAQGVQLTAKLGYPITDDLDIYTRLGGMVWRADTK**SNVYG**KNHDTGVSPVFAGGVEYAITPEIATRLEYQWTNNIGDA**H**TIGTRPDNGMLSLGVSYRFGQGEAAPVV**A**PAPAPAPEVQTKHFTLKSDVLF**N**FNKATLKPEGQAALDQLYSQLSNLDPKDGSVVVLGYTDRIGSDAYNQ**G**LSERRAQSVVDYLISKGIP**A**DKISARGMGESNPVTGNTCDNVKQRAALIDCLAPDRRVEIEVKGIKDVVTQPQA

>*S. boydii* str. Sb227 (II, alpha)

MKKTAIAIAVALAGFATVAQAAPKDNTWYTGAKLGWSQYHDTGFI**P**NNGPTHENQLGAGAFGGYQVNPYVGFEMGYDWLGRMPYKG**DNI**NGAYKAQGVQLTAKLGYPITDDLDIYTRLGGMVWRADTK**ANVPGGASF**KDHDTGVSPVFAGGVEYAITPEIATRLEYQWTNNIGDA**N**TIGTRPDNGLLSLGVSYRFGQGEAAPVV**A**PAPAPEVQTKHFTLKSDVLF**N**FNKATLKPEGQAALDQLYSQLSNLDPKDGSVVVLGYTDRIGSDAYNQ**G**LSERRAQSVVDYLISKGIP**A**DKISARGMGESNPVTGNTCDNVKQRAALIDCLAPDRRVEIEVKGIKDVVTQPQA

>*S. boydii* str. 965-58 (V, alpha)

MKKTAIAIAVALAGFATVAQAAPKDNTWYTGAKLGWSQYHDTGFI**D**NNGPTHENQLGAGAFGGYQVNPYVGFEMGYDWLGRMPYKG**SVE**NGAYKAQGVQLTAKLGYPITDDLDVYTRLGGMVWRADTK**AHNNVTGESE**KNHDTGVSPVFAGGVEWAITPEIATRLEYQWTNNIGDA**H**TIGTRPDNGLLSLGVSYRFGQGEAAPVV**A**PAPAPAPEVQTKHFTLKSDVLF**N**FNKATLKPEGQAALDQLYSQLSNLDPKDGSVVVLGYTDRIGSDAYNQ**G**LSERRAQSVVDYLISKGIP**A**DKISARGIGESNPVTGNTCDNVKQRAALIDCLAPDRRVEIEVKGIKDVVTQPQA

>*S. flexneri* str. 8401 (II, alpha)

MKKTAIAIAVALAGFATVAQAAPKDNTWYTGAKLGWSQYHDTGFI**P**NNGPTHENQLGAGAFGGYQVNPYVGFEMGYDWLGRMPYKG**DNI**NGAYKAQGVQLTAKLGYPITDDLDIYTRLGGMVWRADTK**ANVPGGASF**KDHDTGVSPVFAGGVEYAITPEIATRLEYQWTNNIGDA**N**TIGTRPDNGLLSLGVSYRFGQGEAAPVV**A**PAPAPEVQTKHFTLKSDVLF**N**FNKATLKPEGQAALDQLYSQLSNLDPKDGSVVVLGYTDRIGSDAYNQ**G**LSERRAQSVVDYLISKGIP**A**DKISARGMGESNPVTGNTCDNVKRRAALIDCLAPDRRVEIEVKGIKDVVTQPQA

>*S. flexneri* str. 1485-80 (I, alpha)

MKKTAIAIAVALAGFATVAQAAPKDNTWYTGAKLGWSQYHDTGFI**N**NNGPTHENQLGAGAFGGYQVNPYVGFEMGYDWLGRMPYKG**SVE**NGAYKAQGVQLTAKLGYPITDDLDIYTRLGGMVWRADTK**SNVYG**KNHDTGVSPVFAGGVEYAITPEIATRLEYQWTNNIGDA**H**TIGTRPDNGMLSLGVSYRFGQGEAAPVV**A**PAPAPAPEVQTKHFTLKSDVLF**N**FNKATLKPEGQAALDQLYSQLSNLDPKDGSVVVLGYTDRIGSDAYNQ**G**LSERRAQSVVDYLISKGIP**A**DKISARGMGESNPVTGNTCDNVKQRAALIDCLAPDRRVEIEVKGIKDVVTQPQA

>*Y. pestis* (other, other)

MKKTAIALAVALVGFATVAQAAPKDNTWYTGGKLGWSQYQDTGSI**I**NNDGPTHKDQLGAGAFFGYQANQY

LGFEMGYDWLGRMPYKG**DIN**NGAFKAQGVQLAAKLSYPVAQDLDVYTRLGGLVWRADAK**GSFDGGLDRA**S

GHDTGVSPLVALGAEYAWTKNWATRMEYQWVNNIGDR**E**TVGARPDNGLLSVGVSYRFGQE**D**AAAPIVAPT

PAPAPIVDTKRFTLKSDVLF**G**FNKANLKPEGQQALDQLYAQLSSIDPKDGSVVVLGFADRIGQPAPNL**A**L

SQRRADSVRDYLVSKGIP**A**DKITARGEGQANPVTGNTCDNVKPRAALIECLAPDRRVEIEVKGYKEVVTQ

PQA

>*K. pneumonia* (other, other)

MKKTAIAIAVALAGFATVAQAAPKDNTWYAGGKLGWSQYHDTGFY**G**NGFQNNNGPTRNDQLGAGAFGGYQ

VNPYLGFEMGYDWLGRMAYKG**SVD**NGAFKAQGVQLTAKLGYPITDDLDIYTRLGGMVWRADSK**GNYASTG**

**VSR**SEHDTGVSPVFAGGVEWAVTRDIATRLEYQWVNNIGDA**G**TVGTRPDNGMLSLGVSYRFGQEDAAPVV

**A**PAPAPAPEVATKHFTLKSDVLF**N**FNKATLKPEGQQALDQLYTQLSNMDPKDGSAVVLGYTDRIGSEAYN

Q**Q**LSEKRAQSVVDYLVAKGIP**A**GKISARGMGESNPVTGNTCDNVKARAALIDCLAPDRRVEIEVKGYKEV

VTQPAA

>*R. planticola* (other, other)

MKKTAIAIAVALAGFATVAQAAPKDNTWYAGGKLGWSQYHDTGFY**G**NGFQNNNGPTRNDQLGAGAFGGYQ

VNPYLGFEMGYDWLGRMAYKG**SVD**NGAFKAQGVQLTAKLGYPITDDLDIYTRLGGMVWRADADGNYGSTG

VSRSEHDTGVSPVFAGGLEWAVTRDIATRLEYQWVNNIGDA**G**TVGARPDNGMLSLGVSYRFGQEEAAPVV

**A**PAPAPAPEVTTKHFTLKSDVLF**N**FNKSTLKPEGQQALDQLYTQLSNMDPKDGSAVVLGYTDRIGSDAYN

Q**Q**LSEKRAQSVVDYLVSKGIP**A**GKISARGMGESNPVTGNTCDNVKARAALIDCLGPDRRVEIEVKGYKDV

VTQPQA
